# Chinese Consumers’ Preferences for Attributes of Fresh Milk: A Best–Worst Approach

**DOI:** 10.3390/ijerph16214286

**Published:** 2019-11-05

**Authors:** Shaosheng Jin, Rao Yuan, Yan Zhang, Xin Jin

**Affiliations:** 1 China Academy for Rural Development, School of Public Affairs (CARD), Zhejiang University, Hangzhou 310000, China; ssjin@zju.edu.cn (S.J.); xinj@zju.edu.cn (X.J.); 2 Department of Agricultural Economics and Management, School of Public Affairs, Zhejiang University, Hangzhou 310000, China; 11922005@zju.edu.cn; 3 State Key Laboratory of Grassland Agro-ecosystems, College of Pastoral Agriculture Science and Technology, Lanzhou University, Lanzhou 730000, China

**Keywords:** fresh milk, attributes, BWS, LCM

## Abstract

Fresh milk represents a major type of dairy product in China, while it remains at a low level in terms of consumption. Consumers’ preferences are a crucial factor determining consumers’ attitudes and behavior towards fresh milk consumption. However, little is known about what drives consumers’ preferences for fresh milk. This study intends to fill this gap based on a survey with a sample of 1248 respondents in ten cities in China. The best–worst scaling (BWS) method was employed to measure the importance of different attributes that consumers consider when selecting fresh milk. The BWS result indicates that safety certification, shelf-life, and nutrition were ranked as the most important attributes of fresh milk, whereas origin, purchasing location, and package were found to be the least preferred attributes. Furthermore, the study also explored heterogeneities by dividing all samples into a developed area and a less developed area, and a latent class model (LCM) was then applied to classify consumers based on their preferences and demographics in these two areas, respectively. In developed areas, consumers were divided into “Safety First” and “Taste Sensitive”, and *Gender* and *Age* were significant determinants of class membership. In less developed areas, consumers were classified as “Quality Sensitive” and “Brand Sensitive”, while *Education*, *Pregnancy*, and *Health condition* were significant determinants of class membership. The findings might be useful for the government in terms of encouraging dairy companies to implement safety management certification, and suggest that companies should design differentiated strategies in different areas.

## 1. Introduction

Dairy and milk consumption is considered a crucial element in a healthy and balanced diet [1]. It provides the energy and nutrients necessary for proper growth and development, being important in respect to preventing several chronic conditions, such as cardiovascular diseases, some forms of cancer, obesity, and diabetes [1,2]. Demand for dairy has been expanding dramatically in China; however, the per capita dairy consumption is low (16.50 kg in 2016), and is far lower than in the EU and America, and even lower than the global average or the average of Asian areas [3]. Given the growing population of China, there is ample room for future growth in dairy product needs in China. 

**Figure 1:**
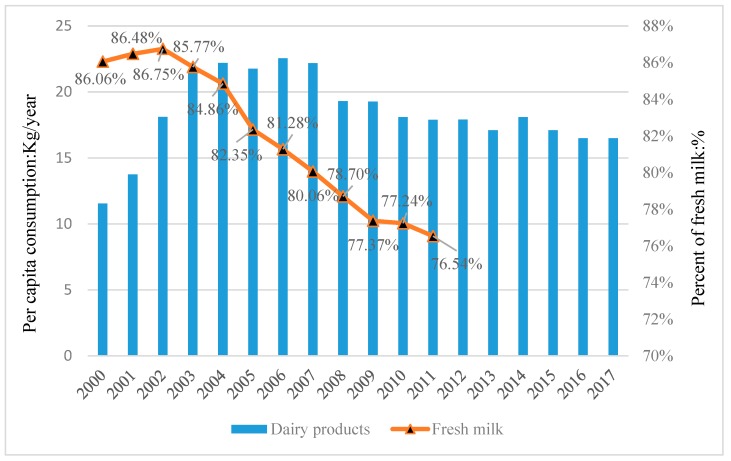
Chinese urban consumers’ per capita dairy consumption. Data Source: China Dairy Yearbook, 2000–2017. Note: Dairy products include fresh milk, milk powder, and yogurt.

Figure 1 shows the dairy consumption and proportion of fresh milk in the total dairy consumption in Chinese urban areas. The per capita consumption of dairy products surged from about 11.55 kg in 2000 to 22.5 kg in 2007, representing an increase of 95.65% [4]. However, it fell to approximately 18 kg after the melamine scandal in 2008, remaining at this level until recent years. Fresh milk is the main consumption type of dairy production among urban consumers. Fresh milk consumption accounted for about 86% of the total dairy consumption in 2000 and 76% in 2011, and remains the main type of dairy consumption in China. 

Previous evidence indicates that many factors can influence the consumption of dairy products, including income, price, preference, education, advertising, marketing channels, and convenience [5,6,7], as well as food safety concerns [8]. These factors can be sorted into three major aspects, including consumers’ characteristics (e.g., preference, gender, age, income, education, etc.), economic factors (e.g., economic growth), and external factors (e.g., food safety incidents). In China, however, the influence of economic factors in dairy consumption should be decreasing, since consumers’ incomes have increased enough for them to be able to afford milk products, especially in the case of urban consumers [7]. Food safety incidents have been identified as having a significant impact on milk consumption [8,9,10,11,12,13]. The Sanlu group melamine-contaminated milk power incident in the milk industry in 2008 rocked China, leading to several hundred thousand infants suffering from procreation and urinary system damage, and the death of four children. Meanwhile, this scandal drove thorough rectification and supervision from the government [12,13]. With the improvement of the quality and safety level of the dairy industry, the impact of the melamine incident is becoming weaker, and has recently had little impact on fresh milk consumption. 

There are many discussions about consumer characteristics, in which the consumers’ preferences have been identified as an important factor [5]. However, whilst the relevant literature has paid attention to assessing consumers’ willingness to pay for a certain attribute of dairy products [9,14,15,16], little is known about current Chinese consumers’ preferences for multiple attributes of fresh milk. Besides, in view of China’s vast territory and regional differences [5,6], it is necessary to consider whether there is heterogeneity in consumer preferences between different cities. 

This study aims to fill these gaps by measuring urban consumers’ preferences towards fresh milk in both developed areas and less-developed areas. The objective of this study is three-fold: first, to measure the importance of attributes that Chinese urban consumers consider when choosing fresh milk; second, to answer what drives consumers’ preferences for fresh milk; and third, to explore whether economics and regional differences influence consumers’ choices of fresh milk. A BWS approach was applied to measure consumers’ preferences for attributes of fresh milk, since it could avoid the shortcomings of a ranking approach (e.g., scale error) and acquire a more authentic result compared with other ranking approaches [17,18,19,20].

The remaining sections of this paper are structured as follows: Section 2 introduces the design of the survey and the models employed in the study, Section 3 discusses the results, and the last section presents the conclusion and implications.

## 2. Methodology 

### 2.1. Survey Design and Data 

#### 2.1.1. Best–Worst Scaling Approach

Many approaches, like rating and ranking, can be used to evaluate the importance or preference for attributes of products or services. For rating, a typical form is the Likert-type scale, where a person responds on a scale of “not important” to “very important” or 1 to 5, with 1 being not important and 5 being very important. However, respondents sometimes do not use ratings in the same way, due to different understandings of the categories [17,18,19]. For example, a 5 for one person could possibly be a 4 for another. Another problem is that the discrepancy between these interval scales is not obvious, so it is hard for respondents to choose [20]. For ranking scales, respondents are required to rank the attributes for specific products or services, so the task is easy to complete when preferences are sought for a small number of attributes. As the number increases, however, the task can become exhausting for respondents. For example, the simplest form of ranking scale is that of “paired comparisons”, developed by Thurstone [21]. Assuming *n* attributes, the number of possible pairs is *n (n-1)/2*, and the respondents have heavy burdens to complete all ranking tasks when *n* becomes large.

The best–worst scaling (BWS) approach, also known as maximum-difference scaling, was developed by Louviere and Woodworth [22] and first published in 1992 [18], and can overcome the limitations presented above. First, respondents only need to choose one most and one least preferred attribute in each choice set. It is relatively easy for respondents to understand and reduces the amount of work for respondents. Furthermore, respondents’ judgment can be better when they only need to evaluate preferences at the extreme, rather than preferences at many levels [23]. Second, the best–worst scaling approach can avoid a scale-use bias [24], and is typically employed when preferences are sought for a large number of attributes. 

The best–worst scaling approach has recently been used in many areas to evaluate people’s preferences for attributes of products or public services. For example, it was used to analyze whether the waiting time length is more important than the quality of care [25], as well as to study the preference of attitudes of consumers towards social and ethical issues [26]. In addition, the best–worst scaling approach was applied to examine people’s evaluation food values in terms of different attributes, such as naturalness, taste, price, safety, and convenience [27]. Currently, it is usually adopted to evaluate consumers’ preference for certain attributes of products [28,29]. Cohen [29] examined people’s preference for wine attributes, such as the grape variety, origin of the wine, brand name, information on the shelf, and so on. Consumers’ preferences for minced pork patties were studied by Jaeger et al. [28] using the best–worst scaling approach. In our paper, it will be used to elicit consumers’ preferences for dairy attributes.

#### 2.1.2. Survey Design

As shown in Table 1, there are 13 attributes of fresh milk. Following Louviere et al. [30], here, we used a balanced incomplete block design (BIBD) to allocate the attributes into the choice sets. The BIBD for *v* attributes is denoted as (*b*, *r*, *k*, λ), where *b* is the number of choice sets (blocks), *r* is the repetition per level, *k* is the number of items in each choice set (block size), and λ is the pair frequency. The design should satisfy the equation, *b* × *k = v* × *r* [30]. Considering the length of the questionnaire and the number of question respondents could easily answer, the design was noted as (13, 1, 1, 1). For 13 attributes, each attribute appears one time across all choice sets, each choice set contains 13 attributes, and each attribute appears once. In addition, before respondents were asked to choose the “best” (most important) and “worst” (least important) attributes, we presented a “cheap talk” to describe the choice situation. The “cheap talk” was defined to standardize the situation and to avoid confusion with special situations where people’s criteria might vary [29].

#### 2.1.3. Data Collection

**Figure 2:**
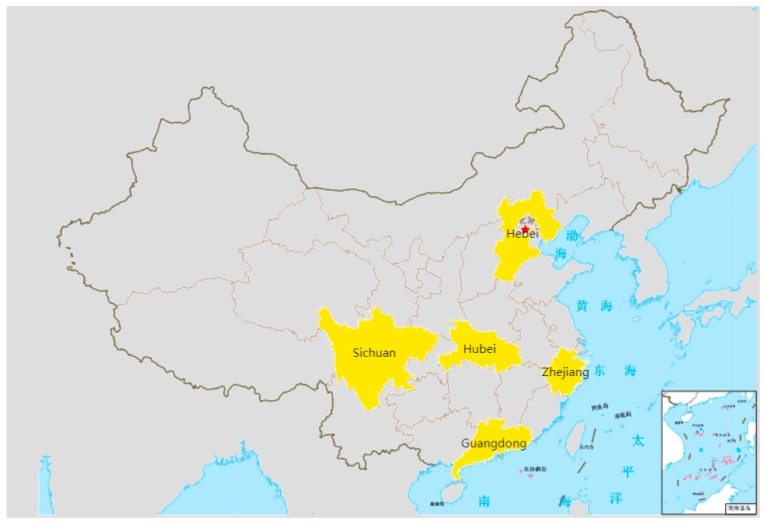
Map of the sample collection sites in China.

Data was obtained from nationwide face-to-face interviews conducted in July and August 2016. Respondents were chosen in a random way. We first selected five provinces, including from the north, south, east, and middle of China. Then, we chose developed cities (capital cities) and less developed cities (non-capital cities) located in these five provinces as the survey cities. They are Shijiazhuang and Chengde in Hebei Province, Guangzhou and Qingyuan in Guangdong Province, Hangzhou and Huzhou in Zhejiang Province, Chengdu and Ziyang in Sichuan Province, and Wuhan and Xianning in Hubei Province (Figure 2). These survey sites were selected to obtain a good representation of China and explore the impact of economic development on consumers’ preference for fresh milk. Before formal interviews, a preliminary test with 18 participants was conducted at a major supermarket in Hangzhou to test the feasibility of the survey design. The procedures of the survey were as follows:

Firstly, a supermarket (e.g., Walmart, Carrefour, CR Vanguard, etc.) in each district of the target city was randomly selected, where actual dairy purchasing decisions take place [31];

Secondly, respondents were recruited, following the principle of randomness and voluntariness (both in the pretest survey and formal survey);

Thirdly, the research assistant introduced the procedure of an interview to ensure that the respondents fully understood the rules of answering the BWS survey, that is, only one most preferred attribute and one least preferred attribute can be chosen; 

Fourthly, introduced the detailed explanation of each attribute; respondent made choice;

Finally, after the BWS question, the respondent finished the rest of the questionnaire. The questionnaire included three sections: the first part included BWS questions, the second part consisted of the demographic characteristics of respondents, and the last part included the consumption habits of dairy products.

### 2.2. Theoretical Model

The BWS choice theory assumes that the probability that a respondent chooses a pair in a particular choice set is proportional to the difference between the ‘best’ and ‘worst’ item on the scale of importance [25]. Respondents are believed to experience the following process: First, identifying all possible pairs. For example, if a choice set has *J* items, or attributes in our case, then there are *J*(*J−1*) possible best–worst pairs that respondents can choose; second, evaluating the difference of importance for every pair. In our case, respondents evaluated the underlying importance of all dairy attributes; Third, choosing the pair that maximizes the difference between the ‘worst’ attribute and the ‘best’ attribute in importance from all *J*(*J*−1) possible pairs [18]. The probability of choosing a given pair is related to the difference on an underlying scale of importance. In other words, it is assumed that respondents will choose the two attributes that have the most differences between each other [23]. Formally, the latent unobserved difference between attributes *b* (best) and *w* (worst) can be defined as

(1) $$D_{bw}=\delta_{bw}+\varepsilon_{bw}$$

where $D_{bw}$ is the measurable difference between attributes *b* and *w* on the underlying scale, and $\varepsilon_{bw}$ is a random error component. The probability of choosing the pair *bw* in the situation *t* is therefore given by

(2) $$P\left(bw/t\right)=P(\delta_{bw}+\varepsilon_{bw}>\delta_{ij}+\varepsilon_{ij})$$

for all *ij* ≠ *bw* in situation *t.* Assuming that $\varepsilon_{bw}$ is a distributed *i.i.d*. type I extreme value, then this probability takes the familiar multinomial logit (MNL) form:

(3) $$P\left(bw/t\right)=\frac{\exp\left(\delta_{bw}\right)}{\sum_{ij}\exp\left(\delta_{ij}\right)}$$

for all *ij* ≠ *bw* in situation *t.* Changing the form of observable difference $\delta_{bw}$ as the difference between two locations (*bw*) on the scale of importance, 

(4) $$\delta_{bw=}\beta_{b}-\beta_{w}.$$

The choice probability is then expressed as

(5) $$P\left(bw/t\right)=\frac{\exp\left(\beta_{b}-\beta_{w}\right)}{\sum_{ij}\exp\left(\beta_{i}-\beta_{j}\right)}$$

for all *ij* ≠ *bw* in *t.* In the estimation procedure, a reference location must be defined from which other items will be evaluated. The β value for the reference item is set to zero, i.e., the reference item is simply removed from the estimations. The dairy attributes are estimated relative to one attribute of reference.

#### 2.2.1. Share of Preference 

It is presumed that consumers are heterogeneous and have different preferences. The logit form of the probability can be further specified using the Random Parameter Logit (RPL) model, which is widely used in the literature and acknowledges the preference heterogeneity of consumers [27,32,33]. Specifically, the importance of parameters for individuals *p* and attributes *j* can be specified as

(6) $$\tilde{\beta_{pj}}=\bar{\beta_{j}}+\sigma_{j}\mu_{pj}$$

where $\bar{\beta_{j}}$ and $\sigma_{j}$ are the mean and standard deviation of $\beta_{j}$ in the population, respectively, and $\mu_{p}$ is a random term normally distributed with a mean of zero and unit of standard deviation. Substituting (6) into Equation (5) yields a probability statement that depends on the random term in $\mu_{pj}$. 

Since there is no natural interpretation of results from the MNL and RPL models, following Lusk and Briggeman [27], we used the ‘share of preference’ to evaluate the importance of each dairy attribute, which is the forecasted probability that each attribute is picked as the most important. The share of preference for attribute *i* can be defined as 

(7) $$share\;of\;preference_{i}=\frac{e^{{\hat{\delta }}^{i}}}{\sum_{i=1}^{J}e^{{\hat{\delta }}^{j}}}$$

These shares of preference must sum to one across all 13 attributes. Equation (7) reports the importance of the attribute *i* on a ration scale, meaning that if one attribute has a shared value that is twice that of another attribute, it can accurately be said that the former attribute is twice as important as the latter [27]. In a word, the share of preference calculations conveys a key message—the probability that an attribute is picked as being more important than others. 

#### 2.2.2. Latent Class Model 

Two major choice models are usually applied to model individual respondent differences for BWS data. One is the RPL model introduced above, which assumes that respondents’ preferences differ along a continuum and estimates a mean and variance for each attribute. The other is the latent class model (LCM), which assumes that there are unique segments (latent classes) of consumers, who have similar preferences within segments, but significantly differ in their preferences across segments. LCM used in the study can predict and explain heterogeneity in preferences that we cannot directly observe [34,35], which is suitable for BWS data since we cannot make a direct observation of an individual consumer’s preferences for a variety of attributes [36].

Therefore, LCM was applied to obtain further insights into the nature of the heterogeneity in respondents’ preferences for attributes of fresh milk. LCM analysis is a clustering technique which assumes that individuals belong to one of k latent classes, of which the size and number are unknown a priori to the researcher [34]. However, unlike other approaches, such as K-means cluster analysis, the probability of class membership is estimated using model parameters and observed covariates, in this case, age, education, income, gender, and so on. LCM has been proven to be suitable for BWS data [37]. In our case, the LCM results provided information on the preference of fresh milk across the sample, as well as data on how demographic variables affect the classification of respondents.

As the choice sets can be different in particular applications, we use $y_{nt}$ to depict a specific choice made by a respondent n as the best attribute, so that

(8) $$\left(j\right)=P\left(y_{nt}=j|\mathrm{class}=q\right),\;q=(1,2,3\ldots ,Q).$$

According to Greene and Hensher [38], we changed the form of Equation (3) to $P_{nt|q}$, while the prior probability for class q for respondent n is denoted as $H_{nq}$. For our case, the multinomial logit with the following form can be considered:

(9) $$H_{nq}=\frac{\exp\left({\overset{´}{z}}_{n}\theta_{q}\right)}{\sum_{q=1}^{Q}\exp\left({\overset{´}{z}}_{n}\theta_{q}\right)},\;q=\left(1,2,3\ldots ,Q\right),\;$$

where *q* is the number of classes and *Z_i_* indicates individual characteristics [39]. The likelihood of this classification and selection for the sample can be expressed as [40]

(10) $$\ln L=\sum_{n=1}^{N}\ln P_{n}=\sum_{n=1}^{N}\ln\sum_{q=1}^{Q}H_{nq}\left(\prod_{t=1}^{T_{n}}P_{nt|t}\right).\;$$

#### 2.2.3. Counting Scores

Finn and Louviere [18] showed that a simpler method for exploiting BWS data leads to results similar to those obtained with logit models. The method involves a series of counting *No. B* and *W*, for each attribute, representing the number of times it was picked as ‘most important’ minus the number of times it was picked as ‘least important’. 

Following Cohen [29], we used the average *(B−W)*, *sqrt(B/W)*, and relative *sqrt(B/W)* to analyze attribute importance, in which the relative *sqrt(B/W)* means that the square root of *(B/W)* for all attributes *(sqrt(B/W)* is scaled by a factor such that the most important attribute with the highest *sqrt(B/W)* becomes 100. All attributes can then be compared to each other by their relative sqrt *(B/W)* ratio. The result is interpreted as *X* per cent (e.g., 60 per cent) being likely to be chosen as the most important. The average *(B − W)* can be expressed as $\;\frac{B-W}{N\times r}$, where n means the number of respondents and r means the frequency with which each attribute appeared in the BW design (one in our case).

## 3. Results and Discussion

Overall, 1248 consumers from 10 cities of five provinces were interviewed. We divided all respondents into two sub-samples: those from five developed cities which have a higher GDP, and those from five less developed cities. The summary statistics for samples are given in Table 2. The average age was about 35 years in the two sub-samples. Females were more numerous than males, which is consistent with the national statistics. Differences have been shown to exist in the two groups with respect to gender, marriage, income, health condition, and number of children in the family, since the *p*-value of the *t*-test was significant.

Table 3 shows the counting results of BWS. According to the average BW score, the top three important attributes were shelf life, safety certification, and nutrition. Purchasing location, package, and origin were the three least preferred attributes. Column 6 is the square root of the best/worst score, and these values are scaled by the most important attribute, which is assigned the highest index with an interval scale of 100. The relative importance shown in column (7) was computed relative to this value. The rankings of relative importance of attributes based on column (7) are shown in column (8). The most important attributes were found to be safety certification, followed by shelf life and nutrition, which is slightly different to the ranking of average BW score. Compared with safety certification (100%), shelf life and nutrition were ranked as the second and third preferred attributes, with a score of 83.83% and 46.77%, respectively. However, other attributes had a low proportion under 20% compared with the score of 100%, which means that Chinese consumers show less interest in these attributes of fresh milk. Take price as an example, which is only 0.05 times as important as safety certification. In accordance with previous studies [27,41,42], the safety attribute was the most important factor in making a purchase decision. However, our results update the previous conclusion, which found that purchasing location and brand were the two most preferred attributes for consumers in a study conducted before 2008 [3]. The discrepancy between these two results may be attributable to the food safety scandals.

### 3.1. Relative Importance of Attributes

Table 4 reports the relative importance of attributes based on the coefficients of the random parameter logit and multinomial logit estimation according to Equation (7). P (> chi2) of MNL model and RPL model are both 0.000, which indicates overall good fitness of the two models. The importance of each attribute was estimated relative to the attribute “Taste” (which was omitted as a benchmark attribute in the regression). The results found that shelf life, safety certification, and nutrition were the top three preferred attributes, followed by brand and organic, although they were insignificant. The remaining attributes, including taste, fat, sterilization technique, traceability, price, purchasing location, and package, seemed to be less important, which is in accordance with the counting results discussed above. Moreover, consumers’ heterogeneous preferences have been confirmed by the RPL model. To ease interpretation of the estimation results, following Lusk et al. [27], we converted the coefficients to the ‘share of preference’, producing ration-scaled scores that sum to 100.

The last column in Table 4 reports the share of preference for each attribute calculated using Equation (7) from RPL, which showed that about 31.3% of people would rate shelf life as the most important attribute of fresh milk, followed by safety certification, with a proportion of 30.2%. Although nutrition was ranked third, shelf life and safety certification are about three times as important (12.6%). Less than 5% of consumers would choose the remaining dairy attributes, including organic, fat, sterilization technique, traceability, price, purchasing location, and package, as the most important attribute. Nevertheless, this shows that brand and organic are over four times more preferred than origin and purchasing location, and more than ten times preferred in comparison to package. These results essentially mean that Chinese consumers in urban areas are mainly concerned about safety certification when they buy fresh milk, and shelf life is also a basic indicative factor used to assess the quality and safety of fresh milk.

One interesting finding in our results was that price was perceived as one of the least important attributes, which was not consistent with the previous literature [27]. BW scores for both sub-samples (shown in Appendix A) also confirmed that, relative importance of price is just about 5%. The possible reason might be due to the economic growth, the changes in dietary pattern especially in urban area, concerning about food safety and nutrition attributes more than price, etc.

### 3.2. Consumer Heterogeneities for Preference of Fresh Milk

In this paper, consumers from developed areas and less developed areas exhibit differences in terms of demographics, as shown in Table 2. Although they have similar preferences in terms of the relative importance for fresh milk attributes, the literature has shown that dairy consumption displays significant disparities across regions [5,6]. This is likely because of differences in diet habits and the economic environment. Therefore, a further analysis of preference heterogeneity was conducted to search for the presence of potential clusters of respondents with homogeneous preferences.

LCM was adopted to explore the preference heterogeneities for attributes of fresh milk in the two groups, i.e., the capital cities and non-capital cities. Based on Latent Gold choice 5.0 software, we observed two clusters in both regions based on Bayesian Information Criteria (BIC), shown in Table 5, which had the smallest BIC value (A smaller BIC implies a better estimation. Furthermore, we estimated the robust standard errors and Wald statistics to validate our results) [43]. The clustering results are given in Table 6 and Table 7.

Table 6 shows the results of the sub-sample from a capital city. In order to generalize the features of these two clusters, we named cluster 1 as “Safety First”, in which respondents tend to focus on safety certification, shelf life, and nutrition, and pay less attention to purchasing location and origin attribute. The second cluster is labeled as “Taste Sensitive” due to the high importance of the taste attribute compared with the “Safety First” cluster. Considering the social-demographic factors, gender and age are significant determinants of cluster membership. Consumers who are female and older have a higher probability of being “Safety First” members. One possible explanation for this is that females are mainly responsible for the diet of the family and accumulate knowledge on food safety certifications in China, so they pay much attention to safety certification when buying fresh milk. Elder consumers might focus more on their health condition, so they put more emphasis on the safety and nutrition attributes. In contrast, consumers who are male and young tend to be “Taste Sensitive”, probably because young people and males have a strong sense of taste, and also are not the major food buyers of their family, so the taste attribute is considered an indicator of a purchase decision.

However, 78.70% of the sample were placed in the “Safety First” cluster, while just 21.30% were placed in the “Taste Sensitive” group, which indicates that most of the consumers in developed cities pay more attention to safety attributes of fresh milk.

Table 7 shows the LCM results of the sub-sample from non-capital cities. According to the cluster analysis, two clusters, i.e., “Quality Sensitive” and “Brand Sensitive”, were generated, representing 46.55% and 53.45% of the sample, respectively. With respect to the influencing factors, education, pregnancy, and health condition all have a significant impact on clustering.

In the “Quality Sensitive” cluster, respondents gave a distinctively high rating for safety certification, shelf life, taste, and nutrition attributes, and price also played an important role. That is to say, these consumers not only pay attention to quality attributes, but also place some attention on price. The characteristics of consumers in this cluster tend to be those with a lower education, a pregnant family member, or a perceived bad health condition. This is probably because they are vulnerable groups. It is common sense that pregnant women tend to pay more attention to their daily diet, and have stringent requirements for their food safety and nutrition levels, as well as taste. Meanwhile, consumers without a good health condition might attach more importance to food safety and nutrition as well. The second cluster was called “Brand Sensitive”, since this type of consumer has a relatively high score for brand compared to cluster 1. Although safety certification and shelf life have a larger score than brand, they did not show many differences. Consumers in the “Brand Sensitive” cluster tend to have a higher level of education, no pregnant women in the family, and a good situation in terms of health. Conversely, in less developed areas, a local brand dominates the fresh milk markets, whilst a national brand has a lower market share. A possible reason for this might due to the less developed cold chain logistics and inconvenient transportation. Although the size of the sample in these two clusters shows no large difference, with values of 46.55% and 53.45%, respectively, more actions should be taken by both the government and companies to meet consumer demands, such as the construction of logistics infrastructure and development of brands.

Overall, we can see that some differences exist in terms of dairy consumption in developed cities and less developed cities. In developed cities, consumers were divided into two clusters: “Safety First” and “Taste Sensitive” clusters. Comparatively, in less developed cities, consumers were classified as “Quality Sensitive” and “Brand Sensitive”. A large population of consumers in capital cities tended to be in the “Safety First” cluster. However, in less developed cities, there was not a large difference in the size of the sample between “Quality First” and “Safety Sensitive”.

## 4. Conclusions

Recently, the food consumption pattern in China has shifted from grains to higher-protein products such as dairy products, especially in urban areas. The dairy demand has exhibited significant growth and has ample space to increase [44]. However, there has been no significant growth of fresh milk consumption in recent years in urban areas of China. Based on a sample of 1248 consumers in ten cities of China, we explored Chinese urban consumers’ preferences for fresh milk by using a best–worst scaling approach.

Firstly, the BWS score shows that safety certification, shelf life, and nutrition are considered the most preferred attributes, followed by taste, brand, organic, fat, sterilization technique, traceability, and price. However, origin, purchasing location, and package are perceived to be the least important attributes. These results indicate that safety certification, nutrition, and freshness play an important role in consumers’ purchase decision. Therefore, the regulation authority and dairy industry should spare no effort to encourage dairy companies to adopt safety certification and management to decrease consumer concerns about dairy products. Then, dairy enterprises should place an emphasis on the quality of raw milk and adopt advanced processing technology to retain nutrients. Moreover, in order to keep the freshness of fresh milk, dairy enterprise and the supply chain should develop cold-chain transportation and improve the transportation efficiency, in order to shorten the retail distance, which is also an effective approach. Such measures would provide safety assurance [33] and guarantee the nutrition and freshness of fresh milk, and could thus contribute to the development of the Chinese dairy industry in the long run.

Secondly, the LCM results found that Chinese consumers have heterogeneous preferences for the consumption of fresh milk. In developed areas, most of the consumers can be placed in the “Safety First” cluster, which tends to include elderly and female consumers. By contrast, in less developed areas, about half of the consumers can be placed in the “Quality Sensitive” cluster and a half in the “Brand Sensitive” cluster, and special populations such as pregnant woman and people with bad health conditions tend to be in the “Quality First” group.

Overall, Chinese consumers have consistent preferences for attributes of fresh milk across different regions. However, differences exist in terms of factors influencing consumers’ preferences. This finding has an implication for milk companies, suggesting that they should identify target customers and design marketing strategies combining the fresh milk preferences.

## Figures and Tables

**Table 1:** Description of fresh milk attributes.

No.	Attributes	Definition
1	Taste	The extent to which the consumption of milk is appealing to senses.
2	Origin	Where the dairy fresh milk is produced (e.g., foreign countries and domestic).
3	Safety certification	Safety certification refers to dairy companies’ quality management system certificate, such as HACCP and ISO9001.
4	Price	The price that is paid for the fresh milk.
5	Nutrition	Amount and type of protein, vitamins, etc.
6	Butterfat	The amount of fat in milk.
7	Brand	The brand name.
8	Package	The design style of the fresh milk (e.g., Tetra Pak film bag, sterile pillow, sterile brick, or glass bottle, color or pattern of packaging).
9	Purchasing location	The location where consumers purchase fresh milk (e.g., supermarket, convenience store, vending machine, etc.).
10	Organic	The fresh milk is certificated as organic food.
11	Traceability	Whether people can trace the supply chain and production process.
12	Shelf life	Shelf life in the study refers to the time from consumer purchase to milk spoilage.
13	Sterilization technique	The pasteurization technique. Two major types of pasteurized technique are the Low Temperature Long Time and High Temperature Short Time techniques.

**Table 2:** Description of social-demographic characteristics.

Variables	Description and Measurement	Total Sample (*N* = 1248)		Capital City (*N* = 646)		Non-Capital City (*N* = 602)		Capital vs. Non-Capital *t*-test
		Mean	S.D	Mean	S.D	Mean	S.D	*p*-Value
Gender	1 if Male, 0 otherwise.	0.458	0.498	0.500	0.500	0.420	0.493	0.005
Age	Age of respondent as of 2016.	34.712	12.386	34.710	12.847	34.710	11.881	0.999
Marriage	1 if marriage, 0 if single.	0.630	0.482	0.600	0.491	0.670	0.470	0.006
Education	1 if Primary school or below, 2 if Junior high school, 3 if Senior high school, 4 if Junior college or above.	2.460	0.839	2.680	0.794	2.220	0.822	0.457
Income	Annual income of the respondent (Ten thousand Chinese Yuan).	6.089	7.872	7.369	9.252	4.715	5.750	0.000
Children	1 if there is/are family member(s) under 12 years old.	0.466	0.499	0.040	0.204	0.510	0.500	0.003
Elder	1 if there is/are family member(s) over 60 years old.	0.435	0.496	0.430	0.495	0.450	0.497	0.488
Pregnancy	1 if there is a/are family member(s) who is/are pregnant.	0.050	0.209	0.040	0.204	0.050	0.214	0.683
Health condition	1 if respondent has a good health condition, 0 otherwise.	0.690	0.462	0.670	0.472	0.720	0.451	0.047

**Table 3:** Best–worst scores: attribute importance measures.

Attributes	(1) Total Best	(2) Total Worst	(3) *(B−W)* Scores	(4) Average *(B−W)*	(5) Ranking for (4)	(6) $\sqrt{B/W}$	(7) Relative Importance	(8) Ranking for (7)
Shelf life	389	9	380	0.304	1	6.570	83.83%	2
Safety certification	369	6	363	0.291	2	7.840	100.0%	1
Nutrition	148	11	137	0.110	3	3.670	46.77%	3
Taste	135	66	69	0.055	4	1.430	18.24%	4
Brand	87	78	9	0.007	5	1.060	13.47%	5
Organic	18	18	0	0.000	6	1.000	12.75%	6
Butterfat	6	33	−27	−0.022	7	0.430	5.44%	9
Sterilization technique	21	51	−30	−0.024	8	0.640	8.18%	7
Traceability	21	73	−52	−0.042	9	0.540	6.84%	8
Price	21	121	−100	−0.080	10	0.420	5.31%	10
Origin	23	193	−170	−0.136	11	0.350	4.40%	11
Purchasing location	8	181	−173	−0.139	12	0.210	2.68%	12
Package	2	408	−406	−0.325	13	0.070	0.89%	13

Notes: Column (3) = Columns (1)–(2); Column (4) = Column (3)/ (1248*1); Column (5) = Ranking based on column (4); Column (6) = Square root of column (1)/column (2); Column (7): Standardized square root interval scale, i.e., relative sqrt (B/W); Column (8) = Ranking based on column (7).

**Table 4:** Relative importance of attributes.

MNL			RPL		
Attributes	Coef.	Share of Preference	Coef.	SD	Share of Preference
Shelf life	2.372 ^***^ (0.096)	0.366	1.345 ^***^ (0.137)	1.065 ^***^ (0.281)	0.313
Safety Certification	2.309 ^***^ (0.070)	0.344	1.309 ^***^ (0.136)	−1.029 ^***^ (0.274)	0.302
Nutrition	1.207 ^***^ (0.087)	0.114	0.436 ^***^ (0.154)	−0.630 ^**^ (0.274)	0.126
Taste	-	0.063	-	-	0.082
Brand	0.094 (0.102)	0.038	−0.762 ^***^ (0.142)	1.476 ^***^ (0.108)	0.038
Organic	0.000 (0.102)	0.034	−0.829 ^***^ (0.157)	−0.013 (0.157)	0.036
Butterfat	−0.279 ^***^ (0.101)	0.026	−1.184 ^***^ (0.160)	−0.004 (0.156)	0.025
Sterilization technique	−0.310 ^***^ (0.101)	0.025	−1.217 ^***^ (0.159)	−0.168 (0.273)	0.024
Traceability	−0.525 ^***^ (0.098)	0.020	−1.459 ^***^ (0.160)	0.301 (0.373)	0.019
Price	−0.939 ^***^ (0.092)	0.013	−1.733 ^***^ (0.155)	0.875 ^***^ (0.175)	0.014
Origin	−1.415 ^***^ (0.083)	0.008	−2.015 ^***^ (0.150)	1.266 ^***^ (0.141)	0.011
Purchasing location	−1.433 ^***^ (0.082)	0.008	−2.343 ^***^ (0.162)	−0.495 (0.321)	0.008
Package	−2.467 ^***^ (0.069)	0.003	−3.203 ^***^ (0.146)	−0.443 (0.334)	0.003
Log likelihood	7191.163		6910.122		
Prob. > chi^2^	0.000		0.000		
Pseudo R^2^	0.183		-		
Number of respondents 1248					

Notes: numbers in parenthesis are standard errors. *** significant at 1%; ** significant at 5%. MNL: multinomial logit; RPL: Random Parameter Logit.

**Table 5:** The Bayesian Information Criteria (BIC) value.

	Cluster	Cluster 1	Cluster 2	Cluster 3	Cluster 4	Cluster 5
Capital city	BIC	919.326	885.339	981.078	1068.049	1152.751
Non-capital	BIC	862.804	848.481	930.429	1007.657	1116.117

**Table 6:** Preference heterogeneities in developed cities.

Attributes	Safety First	Taste Sensitive	Wald	*p*-Value
Class size	78.70%	21.30%		
Taste	−0.465	3.619	1059.940	0.000
Origin	−1.127	−1.912	1059.940	0.000
Safety certification	2.033	3.963	1059.940	0.000
Price	−0.795	−1.053	1059.940	0.000
Nutrition	1.696	1.493	1059.940	0.000
Butterfat	−0.317	−0.100	1059.940	0.000
Brand	1.144	−1.732	1059.940	0.000
Package	−2.395	−1.878	1059.940	0.000
Purchasing location	−1.374	−1.790	1059.940	0.000
Organic	−0.198	0.214	1059.940	0.000
Traceability	−0.297	−1.187	1059.940	0.000
Shelf life	2.440	−0.068	1059.940	0.000
Sterilization technique	−0.346	0.432	1059.940	0.000
Covariates				
Gender	−0.209	0.209	5.637	0.018
Age	0.040	−0.040	12.147	0.000
Marriage	−0.081	0.081	0.464	0.500
Education	0.197	−0.197	2.712	0.100
Income	0.001	−0.001	0.012	0.910
Elder	−0.128	0.128	2.592	0.110
Children	0.040	−0.040	0.176	0.670
Pregnancy	0.091	−0.091	0.167	0.680
Health condition	0.208	−0.208	1.709	0.190
Number of respondents	646			

**Table 7:** Preference heterogeneities in less developed cities.

Attributes	Quality Sensitive	Brand Sensitive	Wald	*p*-Value
Class size	53.45%	46.55%		
Taste	5.409	−1.033	652.335	0.000
Origin	−4.555	−0.697	652.335	0.000
Safety certification	5.886	1.870	652.335	0.000
Price	3.380	−1.715	652.335	0.000
Nutrition	4.656	0.785	652.335	0.000
Butterfat	−2.142	−0.076	652.335	0.000
Brand	−3.949	1.103	652.335	0.000
Package	−4.978	−1.709	652.335	0.000
Purchasing location	−4.228	−0.955	652.335	0.000
Organic	0.616	0.531	652.335	0.000
Traceability	−3.058	−0.381	652.335	0.000
Shelf life	5.896	2.230	652.335	0.000
Sterilization technique	−2.933	0.048	652.335	0.000
Covariates				
Gender	0.015	−0.015	0.047	0.830
Age	0.001	−0.001	0.013	0.910
Marriage	−0.176	0.176	2.559	0.110
Education	−0.166	0.166	3.236	0.072
Income	−0.025	0.025	1.758	0.180
Elder	−0.081	0.081	1.656	0.200
Children	0.025	−0.025	0.120	0.730
Pregnancy	0.325	−0.325	3.580	0.058
Health condition	−0.287	0.287	4.227	0.040
Number of respondents	602			

## Appendix A

**Table A1:** BWS Scores in both capital cities and non-capital cities.

	Capital City (*N* = 646)					Non- Capital City (*N* = 602)				
Attributes	Best	Worst	*B*−*W* Score	Sqrt (*B*/*W*)	Relative Importance	Best	Worst	*B*−*W* Score	Sqrt (*B*/*W*)	Relative Importance
Shelflife	182	6	176	5.5076	79.08%	207	3	204	8.3066	88.80%
Safety	194	4	190	6.9642	100.00%	175	2	173	9.3541	100.00%
Nutrition	93	5	88	4.3128	61.93%	55	6	49	3.0277	32.37%
Taste	61	29	32	1.4503	20.83%	74	37	37	1.4142	15.12%
Brand	55	33	22	1.2910	18.54%	32	45	−13	0.8433	9.01%
Organic	5	16	−11	0.5590	8.03%	13	2	11	2.5495	27.26%
Butterfat	3	20	−17	0.3873	5.56%	3	13	−10	0.4804	5.14%
Sterilization technique	11	26	−15	0.6504	9.34%	10	25	−15	0.6325	6.76%
Traceability	15	40	−25	0.6124	8.79%	6	33	−27	0.4264	4.56%
Price	9	53	−44	0.4121	5.92%	12	68	−56	0.4201	4.49%
Origin	13	90	−77	0.3801	5.46%	10	103	−93	0.3116	3.33%
Purchasinglocation	4	96	−92	0.2041	2.93%	4	85	−81	0.2169	2.32%
Package	1	228	−227	0.0662	0.95%	1	180	−179	0.0745	0.80%
